# Allicin: Chemistry and Biological Properties

**DOI:** 10.3390/molecules190812591

**Published:** 2014-08-19

**Authors:** Jan Borlinghaus, Frank Albrecht, Martin C. H. Gruhlke, Ifeanyi D. Nwachukwu, Alan J. Slusarenko

**Affiliations:** 1 Department of Plant Physiology, RWTH Aachen University, 52056 Aachen, Germany; 2 Department of Human Nutritional Sciences, University of Manitoba, Winnipeg, MB R3T2N2, Canada

**Keywords:** garlic (*Allium sativum*), reactive sulfur species, apoptosis, redox, antimicrobial, cancer, root growth

## Abstract

Allicin (diallylthiosulfinate) is a defence molecule from garlic (*Allium sativum* L.) with a broad range of biological activities. Allicin is produced upon tissue damage from the non-proteinogenic amino acid alliin (S-allylcysteine sulfoxide) in a reaction that is catalyzed by the enzyme alliinase. Current understanding of the allicin biosynthetic pathway will be presented in this review. Being a thiosulfinate, allicin is a reactive sulfur species (RSS) and undergoes a redox-reaction with thiol groups in glutathione and proteins that is thought to be essential for its biological activity. Allicin is physiologically active in microbial, plant and mammalian cells. In a dose-dependent manner allicin can inhibit the proliferation of both bacteria and fungi or kill cells outright, including antibiotic-resistant strains like methicillin-resistant *Staphylococcus aureus* (MRSA). Furthermore, in mammalian cell lines, including cancer cells, allicin induces cell-death and inhibits cell proliferation. In plants allicin inhibits seed germination and attenuates root-development. The majority of allicin’s effects are believed to be mediated via redox-dependent mechanisms. In sub-lethal concentrations, allicin has a variety of health-promoting properties, for example cholesterol- and blood pressure-lowering effects that are advantageous for the cardio-vascular system. Clearly, allicin has wide-ranging and interesting applications in medicine and (green) agriculture, hence the detailed discussion of its enormous potential in this review. Taken together, allicin is a fascinating biologically active compound whose properties are a direct consequence of the molecule’s chemistry.

## 1. Introduction

Allicin, a sulfur-containing natural compound with many different biological properties is responsible for the typical smell and taste of freshly cut or crushed garlic. The general, though not entirely accurate, perception of natural products as mild and largely harmless in comparison to their chemically synthesized counterparts, has been suggested as one of the reasons for their growing preference by consumers, as well as their increasingly popular use in medicine and agriculture [1]. While the pharmaceutical industry in recent decades has largely focused on high-throughput biochemical screening programmes for the discovery and development of new drugs, the use of natural products for medicinal and antimicrobial purposes is an ancient practice [2]. For instance, the oldest medical resource available contains evidence of the prophylactic and therapeutic use of hundreds of plants and plant products [3]. Written on tablets of clay in cuneiform ca. 2600 BC, this ancient Mesopotamian compendium includes prescriptions for the use of *Papaver somniferum* (poppy juice), *Glycyrrhiza glabra* (liquorice), *Cedrus* species *etc.* for the treatment of common ailments and parasitic infections. Other ancient texts like the Egyptian *Codex Ebers* (*Ebers Papyrus*), the Greek “magical papyra” and the Chinese *Materia Medica* are also replete with records of the use of plants and plant extracts in medicine [3,4]. While the *Ebers Papyrus* and the “magical papyri” precisely mention the use of extracts from garlic for medicinal purposes, Virgil, the first century BC Roman poet, highlighted their use in treating snake bites in his *Second Idyll* [5], and the famous Greek physician, Hippocrates, described their efficacy in treating pneumonia and in wound healing in his *Corpus Hippocraticum* [6]. Although onions are the most widely used Allium and one of the world’s most consumed foods [4], it is their more pungent relative garlic, widely notorious for its rather feisty interaction with man’s olfactory receptors, that has received greater attention from investigators. From its various uses as a vampire repellent and an antidote for dog bites in older times, to its more recent perinatal recruitment by Greek midwives for the purpose of warding off “the evil eye” in labour rooms [4], garlic’s journey through time and history is as rich as it is colourful. Speculations abound concerning the origin of the cultivation of *Allium*—The genus to which garlic (*Allium sativum*), leek (*A. porrum*), onion (*A. cepa*), chives (*A. schoenoprasum*) and well over 700 other species belong, but there are early documented reports from Afghanistan, Kazakhstan, Kyrgyzstan, Pakistan, Tajikistan, Turkmenistan, Uzbekistan, and Northern Iran [7,8]. While garlic’s culinary, therapeutic and even spiritual value has been recognized and acknowledged for centuries, it was not until 1944 when Cavallito and Bailey [9] isolated and described the properties of allicin, the compound responsible for garlic's characteristic pungent odour, that researchers gained a clearer insight into the chemical wonder carefully packaged by nature in the composite bulbs of this edible Allium, and thus began decades of extensive research on allicin, “the heart of garlic” [10].

## 2. Biosynthesis of Allicin

**Scheme 1:**
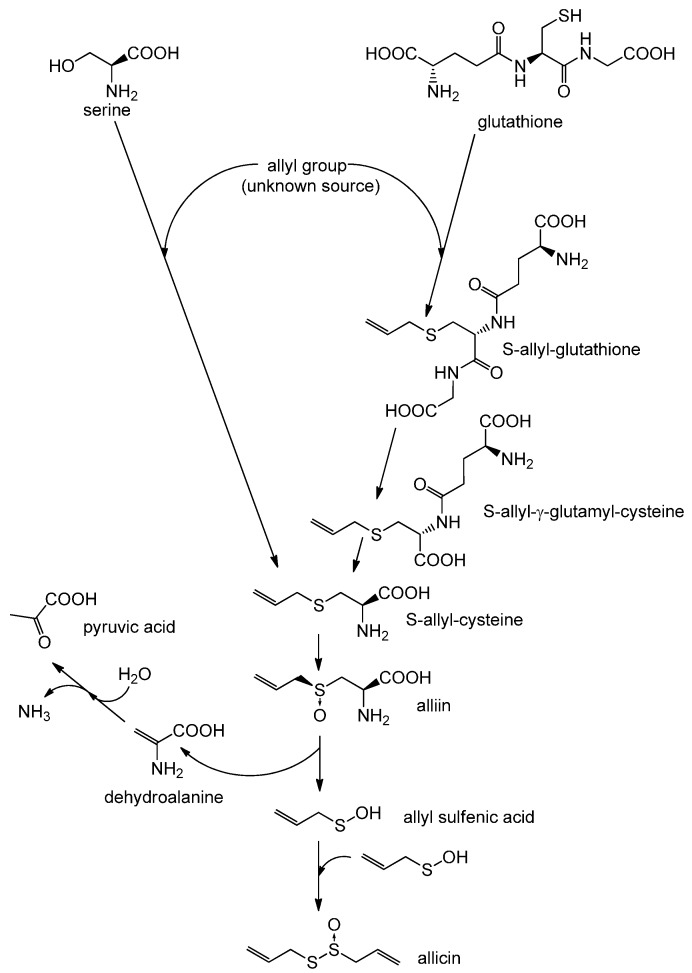
Biosynthesis of allicin: Based on Granroth’s work there are two possible biosynthetic pathways leading to S-allyl-cysteine. The detection of ^14^C-labeled S-allyl-cysteine after feeding plants with ^14^C-labeled serine and the incorporation of various alkyl mercaptans led Granroth to the conclusion that serine is one possible substrate for S-allyl-cysteine biosynthesis. An alternative pathway leads from glutathione to S-allyl-cysteine. This was confirmed by the detection of S-allyl-glutathione and S-allyl-γ-glutamyl-cysteine. The source of the allyl-group is still unknown. S-allyl-cysteine is oxidised to alliin, which is the “inactive” precursor of allicin. Alliin is enzymatically hydrolysed to produce allyl sulfenic acid which condenses spontaneously to allicin.

Allicin is a thiosulfinate, and its structure was determined by Stoll and Seebeck in 1948 [11]. In nature allicin is produced after damage of the plant tissue by an enzymatic reaction. The precursor of allicin is the non-proteinogenic amino acid alliin (S-allyl-l-cysteine sulfoxide) [12]. Alliin and other S-alkyl-l-cysteine sulfoxides are hydrolysed by the enzyme alliinase [13,14], and in the case of alliin this reaction leads to production of dehydroalanine and allyl sulfenic acid. Two molecules of allyl sulfenic acid condense spontaneously to one molecule of allicin [15]. Alliin is found in garlic (*Allium sativum*) and ramsons (*Allium ursinum*) [11]. Interestingly, onion (*Allium cepa*) does not synthesize alliin, but its isomer isoalliin (trans-(+)-S-(1-propenyl)-l-cysteine sulfoxide) [16]. The biosynthetic route to alliin is still not clear. Pioneering work by Granroth [13], who reported two possible biosynthetic pathways based on radioactive labeling experiments, has up to now not been bettered. His findings are shown in Scheme 1.

**Scheme 2:**
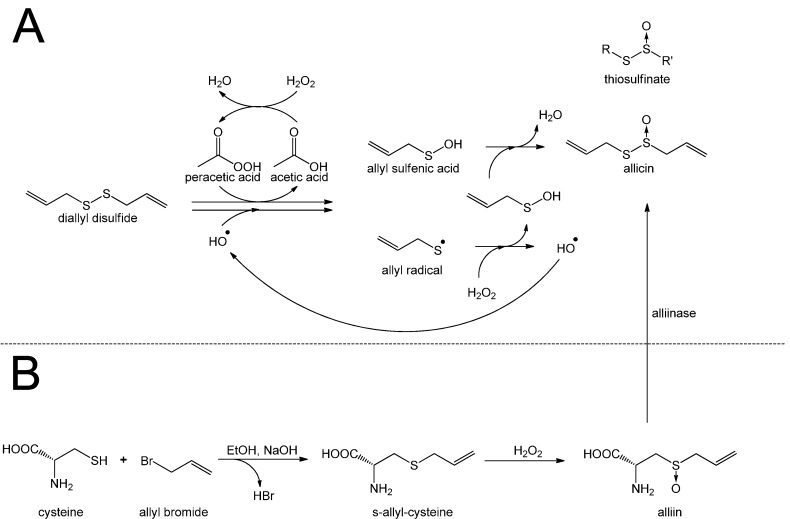
Synthesis of allicin according to Stoll and Seebeck: (**A**) Diallyl disulfide (distilled under reduced pressure) is mixed with acetic acid and hydrogen peroxide. Because hydrogen peroxide reacts very slowly with diallyl disulfide, acetic acid is needed as a catalyst. Peracetic acid (ethaneperoxoic acid) is formed, which is able to oxidize diallyl disulfide to allyl sulfenic acid. This reaction also leads to the production of allyl radicals which can react with hydrogen peroxide to form allyl sulfenic acid and hydroxyl radicals. The latter are able to react with diallyl disulfide to form allyl sulfenic acid and allyl radicals again. Two molecules of allyl sulfenic acid condense to allicin. This reaction mechanism is not only suitable to synthesize allicin but also other thiosulfinates. (**B**) To produce allicin by an enzymatic reaction alliin is needed. Cysteine is mixed with allyl bromide in an alkaline (NaOH) mixture of water and ethanol to obtain S-allyl cysteine. The latter can be oxidized with hydrogen peroxide to produce alliin. By an enzymatic reaction of alliin with alliinase, allicin is formed.

In particular determining the origin of the double bond in alliin is problematic. Granroth was able to show that the double bond in isoalliin originated from methacrylic acid, but this was not the case for alliin, so the source of alliin’s double bond remains unknown. Furthermore, Granroth showed that onion is able to produce alliin when fed with allyl mercaptan or boiled garlic extract. Granroth also showed that not only cysteine but also serine can be a source of the amino acid part of alliin. This was achieved by feeding onion with ^14^C-labeled serine and a variety of thiols. In each case Granroth reported a ^14^C-labeled S-alkyl-l-cysteine sulfoxide, with the alkyl group corresponding to the supplied thiol. In the laboratory allicin can by synthesized by oxidation of diallyl disulfide (DADS) with hydrogen peroxide [17], magnesium monoperoxyphthalate [18] or chloroperbenzoic acid [19]. The oxidation of DADS by hydrogen peroxide is shown in Scheme 2 and reviewed by Ilić *et al.* [15].

Further to the chemical synthesis of allicin, protocols for its enzymatic production *in vitro* have been published [20,21]. The substrate alliin can be extracted from garlic gloves or synthesized from cysteine by alkylation with allyl bromide followed by oxidation with hydrogen peroxide [11,14]. Because of allicin’s high reactivity and low thermal stability it is difficult to obtain and store pure allicin without contamination by related compounds like ajoene, vinyldithiine or polysulfane [22]. Nevertheless, in dilute aqueous solutions at −70 °C preparations have been reported to be stable over years (no loss in two years) [5].

## 3. Redox-Chemistry Pertaining to Allicin

Subcellular compartments need to have regulated but individually diverse conditions to maintain biochemical cellular processes for metabolism. Thus, ionic concentration, pH and an appropriate redox potential need to be carefully maintained. Redox reactions are common in cells and can be recognized because the formal redox state of the atoms in the reactants changes. The concept of redox potential is an aspect of Thermodynamics and the local redox potential will determine whether a particular redox reaction is possible and in which direction a reversible redox reaction can proceed and what equilibrium point it might reach [23,24]. In a biological context many reactions are occurring simultaneously and are present in integrated networks. Thus, although thermodynamic concepts inform us about the possibility, direction and extent of a particular reaction, the picture is incomplete without a consideration of the relative rates of networked reactions, *i.e.*, the Kinetics. For example, a given redox reaction may not be in equilibrium with the redox environment in the cellular compartment because one of the products is rapidly further metabolized or an enzyme catalyzing the reverse reaction proceeds very rapidly whereas the initial reaction is very slow [25]. Nevertheless, the global redox potential in a cellular compartment will influence the reactivity of accessible redox-active cellular metabolites and by this will affect the cell’s chemistry.

In general, healthy cells have a negative cytosolic redox potential—that means the cytosol is in a reduced/reducing state. The cytosolic redox potential of baker’s yeast (*Saccharomyces cerevisiae*) is quoted with values between −220 mV and −320 mV [26]. The cellular redox potential is controlled largely by the GSH/GSSG redox couple (glutathione pool), along with the NAD(P)H/NAD(P)^+^ couples and thioredoxins. Glutathione is a tripeptide consisting of glutamic acid, cysteine and glycine. It was first found by Rey-Pailhade as a biological substance with reducing properties, when he used cell extracts to reduce sulfur to hydrogen sulfide. He named it philothione [27]. Glutathione got its present name from Frederick Hopkins, who, however, thought it to be a dipeptide of glutamic acid and cysteine [28]. Glutathione’s structure was solved by Harington and Mead in 1935 [29]. In cells GSSG is reduced to GSH by glutathione reductase (GR) and NADPH [30,31]. GR is a key enzyme in cellular redox homoeostasis [32,33].

**Scheme 3:**
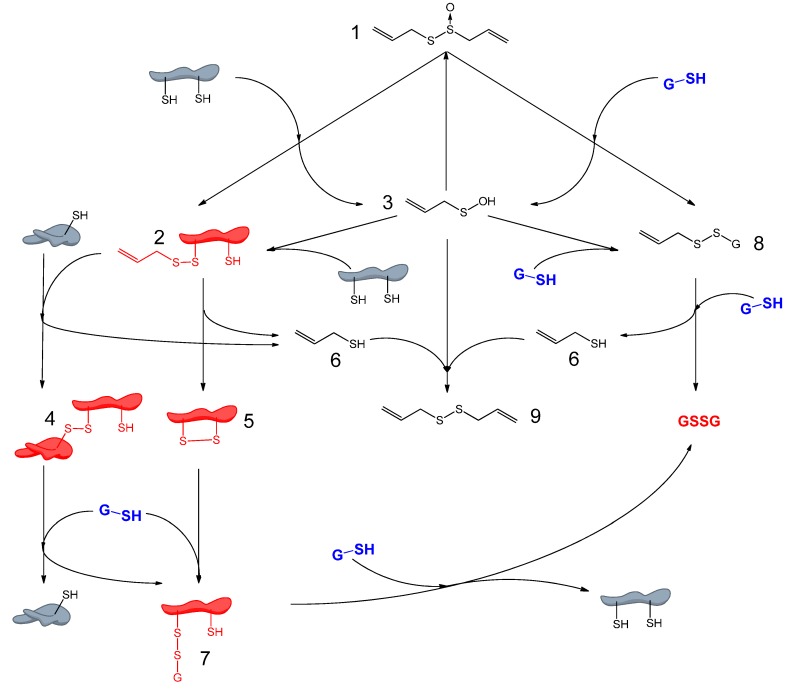
Overview of redox chemistry of allicin and cellular thiols: Allicin (**1**) is able to react with cellular thiols like glutathione (GSH) and cysteine-containing proteins. Reaction with proteins leads to S-allyl-mercapto-proteins (**2**) and allyl sulfenic acid (**3**). S-allyl-mercapto-proteins are able to react with other proteins by formation of disulfide bond-stabilised complexes (**4**) or to form intramolecular disulfide bonds (**5**). Both reactions lead to elimination of allyl mercaptan (**6**). Protein disulfide bonds can be reduced by cellular GSH which leads to S-glutathionyl-mercapto-proteins (**7**). To remove the glutathionyl residues from the proteins another GSH is needed. Allicin also reacts with GSH. This reaction leads to S-allyl-mercapto-glutathione (**8**) and allyl sulfenic acid (**3**). S-allyl-mercapto-glutathione can undergo a thiol/disulfide exchange reaction with another GSH to form GSSG and allyl mercaptan (**6**). Allyl sulfenic acid (**3**), produced in direct reactions of allicin and thiols is able to react with proteins to form S-allyl-mercapto-proteins (**2**), with GSH to form S-allyl-mercapto-glutathione (**8**), with allyl mercaptan (**6**) to DADS (**9**) or with another allyl sulfenic acid (**3**) to form allicin again.

Allicin is a reactive sulfur species (RSS) [23] with oxidizing properties, and it is able to oxidize thiols in cells, e.g., glutathione and cysteine residues in proteins. A more oxidized glutathione pool leads to a higher cellular redox potential. Oxidation of protein thiols can lead to changes in protein structure, for example through disulfide bond formation (for details see Scheme 3). Redox-triggered structural changes in proteins can lead to loss or gain of function. Such effects are already known for the plant protein NPR1, which is a key protein in pathogen-triggered immunity [34] and in yeast (*S. cerevisiae*) for YAP1, which is a redox-sensitive transcription factor coordinating the oxidative stress response [35]. YAP1 is equivalent to the redox regulated mammalian Nrf2/Keap1/ARE system [36]. Detailed investigations to characterize the allicin-dependent redoxome are currently underway in our group.

## 4. Antimicrobial Activity of Allicin

Since ancient times mankind has had to face many different kinds of disease and there was much speculation about their causes. In the Middle Ages the devil turned out to be the usual suspect, however, in the 19th century Robert Koch proved that bacteria caused and transmitted anthrax and the Germ Theory of disease was born. Reports about the targeted use of garlic as an antimicrobial agent go back to the famous Louis Pasteur [37] and in World War I extracts of garlic were used in antibacterial and antiseptic therapeutics. Numerous scientific studies concerning the antibacterial potential of garlic have been published (for a detailed list of reports, refer to Koch and Lawson [5]).

### 4.1. Allicin Is almost Exclusively Responsible for the Antimicrobial Activity of Freshly Crushed Garlic

Isolation and testing of the organosulfur compounds from garlic for antimicrobial activity was carried out in the 1940s [9]. Neither DADS, directly formed by the decomposition of allicin, nor the diallylpolysulfanes showed any remarkable antimicrobial activity, unless used in very high concentrations. Koch and Lawson [5] determined the minimal concentration of organosulfur compounds found in crushed garlic needed to inhibit the growth of *Escherichia coli* and *Staphylococcus aureus*. According to their results, approximately 35× more DADS (6.15 mM) was needed to inhibit the growth of these two bacteria compared to allicin (0.17 mM).

The fact that DADS, as one of allicin’s direct decomposition products, has a significantly lower antimicrobial activity indicates that the thiosulfinate-group (Scheme 2) plays an important role in that activity since it is lost during the reduction of allicin to DADS. Small *et al.* [38] considered thiosulfinates as “*a new class of compounds of which the antibacterial agent of garlic [allicin] represents the prototype*” and chemically synthesized different thiosulfinates, including allicin itself. The thiosulfinate derivatives differed in the alkyl groups that were attached to the thiosulfinate group in chain length as well as in branching. They tested these on twenty different bacterial isolates by elucidating the minimal concentration needed for a bacteriostatic effect in liquid culture. Thereby, two general observations could be made: Firstly, branching of the alkyl groups resulted in a lowered activity. For example, two times more isopropyl thiosulfinate derivative (effective concentrations ranging from 10 to >1200 µM) is needed for a bacteriostatic effect than of the *n*-propyl thiosulfinate derivative (ranging from 10 to 600 µM). Of all the derivatives tested, the most branched alkyl group attached to the thiosulfinate, tert-butyl-ethyl thiosulfinate, showed the weakest activity of all thiosulfinates (ranging from 60 to >1200 µM). The most effective thiosulfinate turned out to be *n*-pentyl-thiolsulfinate (ranging from 0.7 to 130 µM) which was slightly more effective than allicin (ranging from 50 to 150 µM) and furthermore, it was much more stable than allicin due to the lack of double bonds. Secondly, the bacteriostatic effect of thiosulfinates against Gram positive bacteria became stronger with increasing carbon chain length but weaker against Gram negative bacteria.

**Figure 1:**
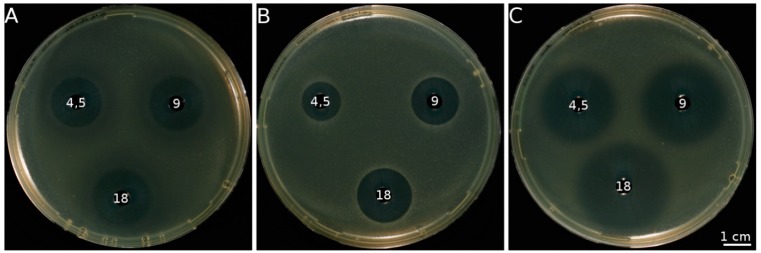
Plate diffusion test. Antibacterial activity against *E. coli* DH5α demonstrated on seeded agar plates by different antibiotics is indicated by clear zones where growth inhibition has occurred. Freshly grown *E. coli* cells (OD_600_ = 0.2, 300 µL) were embedded in 20 mL warm agar in a Petri dish (diameter 9 cm). Afterwards, holes (diameter 0.6 cm) were punched out and filled with 40 µL of antibiotic solution at the mM concentrations indicated. Pictures were taken after 24 h of incubation in the presence of the antibiotics. (**A**) kanamycin; (**B**) allicin; and (**C**) ampicillin.

Depending on the organism and the antibiotic used, on a mol-for-mol basis the effectivity of conventional antibiotics like β-lactams (penicillin and derivatives like ampicillin) or glycosidic antibiotics like kanamycin are comparable to allicin [9,39,40] (Figure 1). However, allicin is active against a broader spectrum of microorganisms than most of the commonly used antibiotics. For example, allicin is active against both Gram positive and Gram negative bacteria, whereas penicillin is practically not effective against the latter [9]. Allicin is also active against human pathogens that are resistant against certain antibiotics. A prominent case of such a germ is methicillin resistant *Staphylococcus aureus* (MRSA)—the chief culprit in many hospital infections. It has been shown that this important pathogen is effectively inhibited by allicin [41].

In several case studies the antimicrobial activity of allicin has been investigated by using garlic extract rather than pure allicin. In general, the antimicrobial activity of garlic extract correlates with the allicin content [42], and if the formation of allicin is inhibited during extraction [12], or if allicin is removed [43], the extract loses its antimicrobial activity. However, Fujisawa *et al.* [40] demonstrated that on a mol-for-mol basis, an allicin-containing extract of garlic was twice as effective as synthetic allicin in inhibiting *Staphylococcus aureus*. These findings indicate either synergistic effects of allicin with other components in the extract, or the additional action of other antimicrobial compounds. Thus, in every case study of allicin activity, the source of allicin (preferably pure allicin) should be taken into consideration. Some bacteria that were tested with either garlic extract and/or pure allicin are listed in Table 1.

### 4.2. A Closer Look at the Basis for Antimicrobial Activity of Allicin

After it was clearly shown by Cavallito and Bailey [9] that allicin is almost exclusively responsible for the antibiotic properties of garlic, the question of the mechanism of allicin’s antibiotic activity arose.

The fact that a compound possesses antimicrobial activity is based on two principal features. Firstly, the compound must be able to reach potential targets and, if these are intracellular, that means it must be able to get inside the microbial cell. In the case of bacteria, an antibiotic has to penetrate the bacterial cell wall and the cell membrane. In addition to these two boundaries, the slime layers or capsules of certain bacteria can constitute an extra layer of resistance [44]. Following the arrival of the antibiotic in the cell, it must have a target that, if attacked, leads to cell inactivity or cell death. Miron *et al.* [45] investigated the permeability of artificial and natural phospholipid membranes to allicin and demonstrated that allicin readily diffuses across these membranes.

**Table 1:** Examples of allicin’s antibacterial activity.

Bacteria	Source of Allicin	Amount of Allicin		Experimental System ^a,b,c,d^	Reference
**Gram positive**					
*Bacillus* spp.	pure allicin extracted from garlic	80 µM		a	[9]
	synthetic	30–150 µM		a	[38]
*Streptococcus* spp.	pure allicin extracted from garlic	80 µM		a	[9]
	synthetic	ranging from 60 µM to 200 µM		a	[38]
methicillin sensitive *Staphylococcus aureus* NBRC 12732	synthetic and garlic extract	2.2 × 10^−3^–0.92 µmol		b, c	[40]
methicillin resistant *Staphylococcus aureus* (clinical isolates)	garlic extract	0.04–0.62 µmol		b, e	[41]
**Gram negative**					
*Salmonella typhimurium*	pure allicin extracted from garlic		80 µM	a	[9]
	enzymatically synthesized from alliin		ranging from 200 µM to 500 µM	a	[46]
*Agrobacterium tumefaciens*	garlic extract		1.72 µmol	b, d	[39]
*Escherichia coli* K12	garlic extract		0.52–1.72 µmol	b, c	[39]
*Pseudomonas syringae* (various pathovars)	garlic extract		1.72 µmol	b, d	[39]
*Vibrio cholerae*	pure allicin extracted from garlic		80 µM	a	[9]

^a^ complete growth inhibition in liquid culture; ^b^ growth inhibition zones; ^c^ via soaked filter discs on top of the seeded agar, ^d^ directly pipetted onto bacteria-seeded agar or ^e^ pipetted into wells punched out of the agar.

When allicin is inside the cell, the antibiotic efficiency depends upon reaching and reacting with its targets and on the importance of those targets to the cell. In a seminal study, using logic that has stood the test of time, Cavallito and coworkers investigated the chemistry of several plant-derived antimicrobial compounds. It was found that the active principles of *Allium sativum*, *Erythronium americanum*, *Asarum reflexum*, *Arctium minus*, *Ranunculus acris*, *Ranunculus bulbosus* and of *Brassica* species as well as the non-plant-derived antibiotics penicillin, citrinin, gliotoxin, clavacin and pyocyanines react with cysteine. Pretreatment of these antibiotics with cysteine led to a total loss of activity against bacteria [47]. The different antibiotics were categorized into groups depending on their reactivity with cysteine residues [48]. The antibiotics were tested with cysteine residues that differed in their chemical microenvironment. In summary, certain antibiotics (group I, e.g., allicin) reacted with every cysteine residue independently of the neighbouring groups, as long as the –SH-group was freely available. Another group of antibiotics (group III, e.g., penicillin) showed increased reaction kinetics with cysteine if other amino-groups were adjacent to the cysteine residue, but much slower reaction kinetics if not. Pyocyanines (group II antibiotics) turned out to be in between, since they showed intermediate reaction kinetics with the cysteines tested. These experiments were prophetically significant considering the present state of knowledge on the effects of surrounding amino acids on the micro p*K*_a_ values (and therefore reactivity) of cysteine residues in proteins to oxidation [25,49]. Furthermore, Cavallito’s group speculated that the lower efficiency of allicin compared to penicillin and other antibiotics could be explained depending on their reactivity with cysteine residues. Since allicin reacts readily with all free cysteine residues available, it is buffered by proteins and low molecular weight thiols, independently of their importance for cellular viability. On the other hand, it was speculated that the specificity of penicillin for cysteine residues in the vicinity of amino-groups would lead to less waste of penicillin on unimportant targets, therefore making it more efficient than allicin [48]. Several case studies have subsequently reported the reactivity of allicin with thiol-containing enzymes [49,50,51]. For example, Wills [52] used allicin to inhibit the activity of several enzymes *in vitro*. Among them were important enzymes for primary metabolism like succinic dehydrogenase, hexokinase, triosephosphate dehydrogenase or alcohol dehydrogenase.

**Figure 2:**
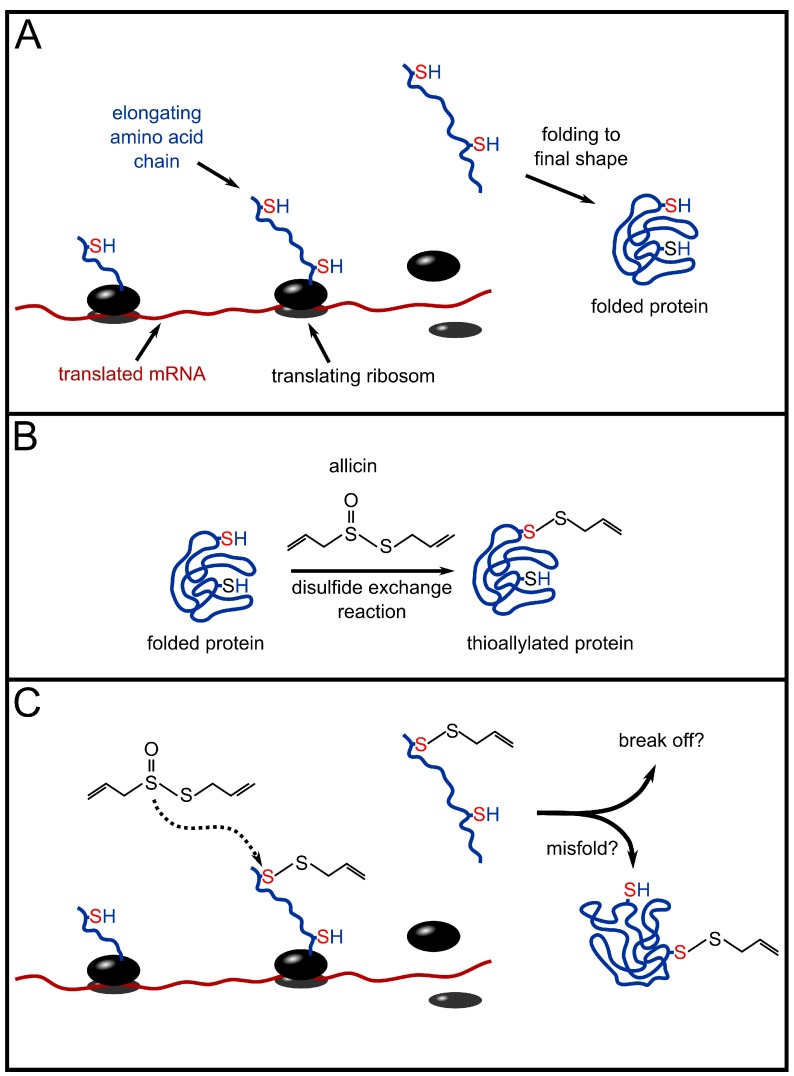
Possible influence of allicin on proteins and protein synthesis. (**A**) protein synthesis in unstressed conditions. After translation the protein is folded into its final structure. (**B**) The cysteine residue that is accessible for attack (indicated in red) reacts with allicin via a disulfide exchange-reaction. The cysteine residue that is sterically blocked (indicated in blue) does not react with allicin. (**C**) According to Cavallito’s hypothesis, allicin may attack cysteine residues on elongating amino acid chains while the protein is still being synthesized and is not fully developed [48]. In this early stage, cysteine residues that are normally blocked for reactions with allicin (compare B) are now potential targets. Possible results may be an abortion of translation or misfolded proteins with reduced or no function.

But it has to be stated that some enzymes which did not contain a thiol group were also inhibited by allicin, whereas a few enzymes containing a thiol group could not. These findings suggest that not only cysteine is a potential target for allicin. The fact that some enzymes, although containing thiol groups, are not inactivated by allicin could be explained by an adverse p*K*_a_ of these cysteines which depends on the microenvironment within the protein [49], so that the reactivity of these cysteines with allicin is very weak. Rabinkov *et al.* [51] investigated the reaction of cysteine with allicin using RP-HPLC and NMR-analysis of the resulting products. It was shown that allicin reacted with the sulfhydryl-group of cysteine via a disulfide exchange-like reaction (Figure 2B, unstressed situation shown in Figure 2A). By demonstrating that enzymes that are irreversibly inhibited by allicin can be rescued by strong reductants, the idea of the disulfide exchange-like reaction was transferred to a working model of enzyme inhibition by allicin via the same mechanism.

### 4.3. Does Allicin have an Effect on DNA-, RNA- and Protein Synthesis?

In 1988, Feldberg *et al.* investigated the effect of allicin on the synthesis of DNA, RNA and proteins in *Salmonella typhimurium* [46]. Here, it was determined that the minimal concentration of allicin needed to inhibit the growth of *S. typhimurium* was 0.3 mM. This allicin concentration turned out to be not lethal, so that *S. typhimurium* recovered and started to grow again at a lower rate, after a lag-period of fifty minutes. He measured the uptake of [^3^H] labeled uridine, leucine and thymidine in allicin-stressed and unstressed control cells and observed a maximum decrease of 68% for [^3^H]-thymidine uptake, a 65% decrease in [^3^H]-leucine uptake and a 100% decrease in [^3^H]-uracil uptake a few minutes after allicin had been applied to the bacteria. Since all these [^3^H] labeled molecules are building blocks for DNA (thymidine)-, RNA (uracil)- and protein (leucine) synthesis, Feldberg concluded that allicin affected the synthesis of these macromolecules, since there seemed to be no demand for the uptake of their precursors. In his study, however, the possibility that proteins involved in the uptake of these precursors could have been affected directly or indirectly was not investigated. For example, transport proteins could have been directly poisoned if they would have an exposed cysteine group that could be affected by allicin. Indirectly, another possibility could be that the energy supply for the transport proteins was not met because enzymes in metabolic pathways had been affected by allicin treatment. Unfortunately, there was no follow-up investigation on these findings, so it remains unclear if the effect observed really showed a direct decrease in DNA-, RNA- and protein synthesis.

### 4.4. Effect of Allicin on Fungi

In the course of evolutionary time garlic plants developed the alliin/alliinase system, responsible for the production of allicin in freshly injured tissue, as a chemical weapon against biotic enemies. Since allicin is produced from a preformed substrate, without further expenditure of cellular energy or metabolism (*i.e.*, passively), upon an attack by a pathogenic microbe or feeding animal, it belongs to the class of so called “phytoanticipins” [53]. In contrast, defence compounds that are produced by a plant *de novo* after an attack, dependent upon the expenditure of cellular energy and usually *de novo* gene expression (*i.e.*, actively), are called phytoalexins (*ibid*). Besides bacteria, many fungal species are potential pathogens that the plant has to cope with.

Beyond its well documented strong antibacterial properties allicin also shows toxic effects towards fungal cells and is able to inhibit spore germination and hyphal growth *in vivo* and *in vitro* [39]. Some efforts have been made to utilize this activity and develop allicin for application in medical therapy and agricultural plant protection [1,54,55].

Allicin showed promising activity both *in vitro* and *in vivo* against many plant-pathogenic fungal species, including pests, which are of economic importance. For example, *Botrytis cinerea, Plectospherella cucumerina*, *Alternaria brassicicola* and *Magnaporthe grisea* were strongly inhibited *in vitro* by allicin in fresh garlic juice in a plate-diffusion assay using spore-seeded agar [39]. In further studies, seed-disinfection turned out to be an efficient and potential application of allicin. It was shown that carrot seeds, infected with *Alternaria* spp., were disinfected by treatment with garlic juice in a degree comparable to a commercial seed-disinfection product [1]. In another assay, germination and seedling-development of pathogen-infested wheat seeds were enhanced by treatment with garlic juice [56]. Thus, allicin seems to have a potential in seed-disinfection programmes both in ecological agriculture and under low-tech conditions (like in developing countries). Since allicin is easy to obtain from freshly damaged garlic tissue, applications in that direction would allow a plant-protection strategy according to the maxim “*grow your own pesticide*”.

Beside this agriculture-orientated application, fungal infections of humans and animals are a focus of interest [57]. Thus, it was claimed that allicin might be the basis of a strategy to treat aspergillosis in the lung, since allicin is highly volatile and thus can be delivered to the lung by inspiration [58]. Allicin can also easily be applied topically to fungal infections of the skin and hence attempts were made to use allicin in the therapy of *Candida*-infections. Interestingly, allicin’s activity was comparable to the frequently used antimycotic agent fluconazole [55]. There is an emerging interest in understanding the molecular basis of allicin’s fungicidal properties. 

Various studies of allicin’s effect on baker’s yeast show that it acts synergistically with other, known fungitoxic substances like copper [59,60,61,62]. Furthermore, synergistic toxicity of allicin with amphotericin B suggest an impact of allicin possibly on fungal plasma membrane [63,64,65].

A form of programmed cell death called apoptosis, where cells employ a genetic suicide programme, has been known for many years in multicellular organisms where it is employed in tissue turn-over and avoids inflammation responses which would usually occur when cells are damaged. Many stimuli can push cells into the apoptotic cell death programme, among others, oxidative stress. Using yeast as a model for apoptosis is a relatively new development, nevertheless, similarities to apoptotic processes in animal cells justify the transferability of the “apoptosis” term from animals to yeast [66]. It was shown that allicin causes an oxidation of glutathione that results in a shift of the cellular redox-potential to a range correlating, according to Schafer and Buettner [67], with the induction of apoptosis [68]. Using both cytological, biochemical and genetic assays confirmed that allicin pushes yeast cells into apoptosis via the “oxidative route” [69].

The global effects of allicin on the yeast transcriptome were reported by Yu *et al.* [69]. From micro-array experiments it was determined that allicin impaired the expression of genes coding for enzymes of amino acid metabolism (methionine, in particular), iron-uptake, the respiratory chain, thiamine-metabolism and proteasomal protein degradation [69]. The authors postulated that these results might be explained by the action of allicin on several different yeast transcription factors (YAP1, MSN2/4, RPN4, SKN7) (*ibid.*).

In summary, allicin shows a broad spectrum of effects on a variety of fungal species which makes a possible application of allicin in medicinal therapy and agriculture an attractive possibility. On the basis of allicin’s effects on *S. cerevisiae*, it is apparent that the redox-effect of allicin seems to be a central, but not exclusive explanation of allicin’s fungicidal activity.

## 5. Allicin’s Effects on Animal and Human Cells

The variety of physiological effects makes allicin important for medicinal applications. Indeed, garlic has been used for centuries because of its therapeutic and health-promoting properties [4]. Studies have linked garlic consumption and the inclusion of garlic oils in the diet with such health-promoting benefits as the selective reduction of triacylglycerol, total- and LDL-cholesterol concentrations without any alteration in HDL-cholesterol levels [70,71,72]. Epidemiological evidence positively correlating garlic consumption with a reduction in the incidence of various types of cancers also abounds [73,74]. The finding that garlic powder has no effect on blood cholesterol level has been reasonably attributed to a possible loss of garlic’s biologically active components, particularly allicin, during preparation [75]. The presence in crushed garlic of various biologically active (degradation) compounds such as polysulfanes and the vinyldithiins, mean that not all health-promoting activities in crude preparations can be unequivocally ascribed to allicin directly. Our focus here will be restricted to only those health-promoting benefits that have been unequivocally demonstrated to be due to allicin’s specific and direct involvement.

### 5.1. Allicin and Cardiovascular Diseases

Cardiovascular disorders are complex, since they are influenced by different factors. Epidemiological studies unearth more and more variables that contribute to the development of cardiovascular problems to different extents [76]. In particular, general oxidative events like the oxidation of the *low-density lipid protein* (LDL) correlate frequently with atherosclerosis [77]. Although allicin is chemically an oxidant, it acts in lower doses as *antioxidant* at the physiological level [78]. This observation can be explained by the fact that mild oxidative conditions induce the expression of so-called phase II detoxifying enzymes, for instance by the activation of redox-sensitive transcription factors and build up protection against further and stronger oxidative insults.

One example for the oxidation of a redox-sensitive transcription factor by the electrophile allicin is the Nrf2/Keap1 system that regulates the expression of various anti-oxidative enzymes (among others of glutathione-biosynthesis). The fact that allicin can induce the Nrf2/Keap1 system has been shown in various studies [79,80]. It needs to be mentioned that the activation of Nrf2 by allicin is not only important in the context of cardiovascular diseases, but also for various other health related events like neurodegenerative diseases. In that context it was shown that allicin attenuates age-related cognitive and memory deficits by activating the Nrf2-system [80].

**Scheme 4:**
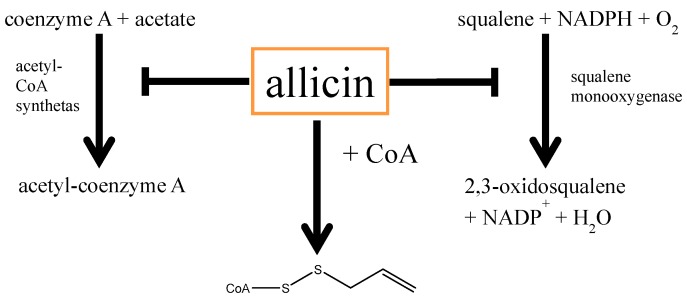
Impact of allicin on cholesterol-metabolism: Allicin was shown to inhibit two important enzymes of the cholesterol-biosynthesis pathway. On the one hand, Gupta and Porter [85] showed that allicin inhibits the squalene-monooxygenase while Focke and coworkers [50] demonstrated an inhibition of acetyl-CoA synthetase, a very early (and thus unspecific) step in cholesterol-biosynthesis. According to its chemistry, one can assume a direct reaction of allicin with the thiol-group of coenzyme A.

According to the so-called “LDL-receptor hypothesis” cholesterol is central for atherosclerosis [81], possibly due to the attraction and activation of macrophages by oxidized LDL, subsequently causing plaques in the arteries. Hence, cholesterol is assumed to be a risk-factor for atherosclerosis and thus for ischaemic disorders like Angina pectoris, cardiac infarction or stroke. One strategy, to interfere with a progression of plaque deposition in arteries is the reduction of the endogenous cholesterol-biosynthesis, commonly by application of statins [82]. While statins in the classical way competitively inhibit the enzyme 3-hydroxy-3-methylglutaryl-coenzyme-A-reductase (HMG-CoA reductase), allicin also shows the ability to suppress cholesterol biosynthesis [83,84], which is ascribed to the inhibition of the squalene-monooxygenase [85] and acetyl-CoA synthetase [50] enzymes. Furthermore, because coenzyme A contains a thiol-group, one can assume that allicin reacts with non-acetylated CoA directly, with the consequence that CoA is not available for biosynthetic processes. This would reduce the biosynthetic rates of CoA-dependent pathways (including sterol-biosynthesis) in a concentration-dependent manner. However, to our knowledge, this *thioallylation* of CoA has not yet been demonstrated. The different effects of allicin on cholesterol-biosynthesis are summarized in Scheme 4.

A further important factor of cardiovascular disorders is platelet-aggregation, which is also important for ischemia of the heart and the brain. Platelet-aggregation is a complex biochemical process. Its prerequisite is the activation of the GPIIb/IIIa receptor by thromboxane A2 which causes the binding of, among others, fibrinogen [86]. Classical platelet-aggregation-inhibitors like acetyl-salicylic acid (aspirin) inhibit endogenous thromboxane-biosynthesis and thus the GPIIb/IIIa receptor activation. Interestingly, thiosulfinates like allicin are potent platelet-aggregation inhibitors. While a final concentration of 0.4 mM allicin inhibits platelet aggregation to about 90%, a comparable concentration of 0.36 mM aspirin shows less than half this activity (~35% inhibition) [87].

A last, but not minor impact of allicin on factors contributing to cardiovascular diseases is hypertension. Allicin acts as an antihypertensive and the reason, again, can be found in allicin’s reactivity. Since allicin decomposes rapidly to its degradation products, it has been shown that a complex reaction cascade with thiols (glutathione in particular) results in the release of hydrogen sulfide (H_2_S) [88] H_2_S, again, is a known potent gaseous signalling molecule in regard to blood-pressure regulation [89]. H_2_S lowers the blood pressure by relaxation of smooth-muscle cells surrounding the blood vessel that can expand and thus results in a lower blood pressure [88].

Taken together, it can be concluded that allicin counteracts cardiovascular diseases in various ways. Because of the high epidemiological impact and prevalence of cardiovascular diseases [90] and due to the fact that allicin is a component of fresh garlic in food [4], allicin and its follow-on products are of great interest influencing cardio-vascular disorders. Although, some aspects are already understood, further efforts are necessary to understand in more detail the molecular basis of allicin’s action. Epidemiological studies addressing the question how the uptake of allicin (or fresh garlic in its natural form) is beneficial for health would also be important.

### 5.2. Allicin’s Immune-Modulatory Activity

Allicin is a strong antimicrobial agent (see above) and is thus, at least *in vitro*, a potent antibiotic. Besides this direct impact on pathogens, a further facet of its activity is the influence on the endogenous immune system. If allicin is able to affect immune-correlated signalling pathways in cells, new possibilities for therapeutic development might be realized: In the case that allicin stimulates the activity of immune cells, this should result in a strengthened defence against pathogens, and suppression of immune processes might be of interest in regard to allergy or auto-immune disorders. In the meantime a plethora of evidence exists suggesting that allicin indeed acts on various immune-correlated processes. 

**Scheme 5:**
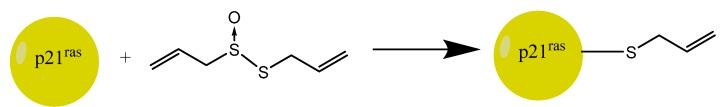
Allicin stimulates lymphocytes by affecting p21^ras^: Thioallylation that means the binding of an allyl sulfenic acid to the thiol of cysteine^118^ leads to an activation of p21^ras^ and subsequently to stimulation of ERK1/2 phosphorylation. These processes are crucial for the activation of lymphocytes.

The initial observation was that allicin inhibits the migration of neutrophilic granulocytes into epithelia, which is a crucial process during inflammation [91]. Anti-inflammatory effects in a general sense have, indeed, been observed, e.g., in the mouse-model of Morbus Bechterew, a degenerative rheumatoid disease of the vertebral body [92]. Furthermore, allicin acts on T-cell lymphocytes by inhibition of the SDF1α-chemokine-induced chemotaxis and this effect is correlated with an impaired dynamic of the actin-cytoskeleton [93]. Finally, it has been shown that allicin inhibits the transendothelial migration of neutrophils (*ibid.*). It should be mentioned that allicin has been shown to have effects on the cytoskeleton in different biological systems. For example, it has been shown in mouse fibroblasts (NIH-3T3) that allicin causes a depolymerisation of the tubulin-cytoskeleton within minutes at a low concentration (2 µM), while with this concentration the actin cytoskeleton remained unaffected [94]. A key player in the activation of lymphocytes is the p21^ras^ protein which triggers the inactivation of the RAS-GTPase by enhancing its enzymatic activity [95]. Interestingly, p21^ras^ appears to be a direct target of allicin since it seems to be thioallylated by allicin and thus activated [96] and, subsequently, this activation might result in an enhanced phosphorylation of the ERK1/2 kinase (*ibid.*), an important MAP-kinase involved in different signalling pathways (Scheme 5).

Another central regulator of a coordinated immune-response is the TNFα cytokine [97]. Impairment of TNFα secretion massively influences the regulation of the immune-response. For that reason it is interesting that allicin inhibits the release of TNFα-dependent pro-inflammatory cytokines in intestinal epithelia [98]. This, however, reflects an effect of allicin on factors downstream of TNFα. Since TNFα is mainly secreted by macrophages [99], the effect of allicin on macrophages was examined with respect to how allicin influences the expression of TNFα itself. It is interesting that a stimulation of macrophages by lipopolysaccharide (LPS) after a pre-incubation in the presence of allicin enhances the activity of the TNFα promoter, suggesting a TNFα-triggering role for allicin in LPS-stimulated cells [100]. Furthermore, allicin inhibits phosphatase-activity, correlating with an enhanced phosphorylation of ERK1/2 (*ibid.*), which is a central component of the signalling cascade transferring extracellular signals into intracellular signalling cascades. Furthermore, the release of Reactive Nitrogen Species (RNS) by LPS-stimulated macrophages was suppressed by allicin [100,101].

It is fascinating if allicin could influence inflammation both directly by exerting an antimicrobial effect and by altering immune-cell signalling. It will be informative to study further the effects of allicin on immune cells on a molecular level and, systematically, it’s impact on the development of inflammatory processes and disease.

### 5.3. Allicin and Cancer

There is a link between the functioning of the immune system and cancer. In an early study (1960), explants of mouse-tumours were incubated in allicin before implantation into healthy mice. In contrast to the control group (where the explants were not allicin-treated), mice with tumour explants incubated in allicin showed no further growth of the explant [102].

How allicin affects cancer cells has been examined at the molecular level. It became clear that the induction of apoptosis was crucial for the anti-cancer effect of allicin. As discussed in the yeast system (see above), allicin also causes a redox-shift in human cell cultures [103]. This leads to the execution of cell death, both in a caspase-dependent [104] and caspase-independent manner [105]. Beside caspase activity, the apoptosis inducing factor (AIF), which contributes to the apoptotic DNA-laddering, is involved in allicin-induced cell death (*ibid.*).

Bat-Chen and co-workers, for instance, showed that Nrf2 is involved in allicin-induced apoptosis [106]. This reveals the “Janus face” of Nrf2 [107]: While Nrf2 is mostly described as an anti-apoptotic factor regulating the expression of anti-apoptotic proteins of the Bcl-2 family like Bcl-2 and Bcl-xL [108,109], Nrf2 also seems to have a pro-apoptotic function under certain circumstances (*ibid.*). Also the ERK1/2 map kinases were shown to be influenced by allicin in immune cells; these kinases are also important for apoptosis induction by allicin [103,110].

Although this paragraph gives only a brief overview about the impact of allicin on cancer cells, some major factors targeted by allicin can be outlined. One problem exacerbating a clinical application of allicin is its chemical instability. As soon allicin is taken up by the body, or at the latest in the circulatory system, it will react with accessible thiols and in particular with the high amount of glutathione and also be decomposed to other compounds. This makes an application as a *pharmaceutical* at the moment unlikely. However, the potential for using garlic in a *nutriceutical* context, with health benefits not only for cancer prevention or therapy, but also for the other medical areas mentioned here, remains an attractive possibility. A sophisticated attempt to circumvent the stability problem is a coupling of alliinase to a delivery system and supplying the stable substrate alliin, thus allowing production of allicin *in situ* at the position of the particular epitope. In this regard, attaching alliinase via antibodies against cancer cells successfully showed a promising proof-of principle [111].

## 6. Effects of Allicin on Plants

### Effects on Germination, Root Growth and Viability on Arabidopsis thaliana

Having already discussed allicin’s effects on bacteria, fungi and animal systems in this review it remains to comment on the effects of allicin on plant physiology. In our lab we investigated the effects of allicin treatment on different developmental stages of the model plant *Arabidopsis thaliana*. The effect of increasing allicin concentrations on the development of Arabidopsis (accession Col-0) seedlings was monitored.

**Figure 3:**
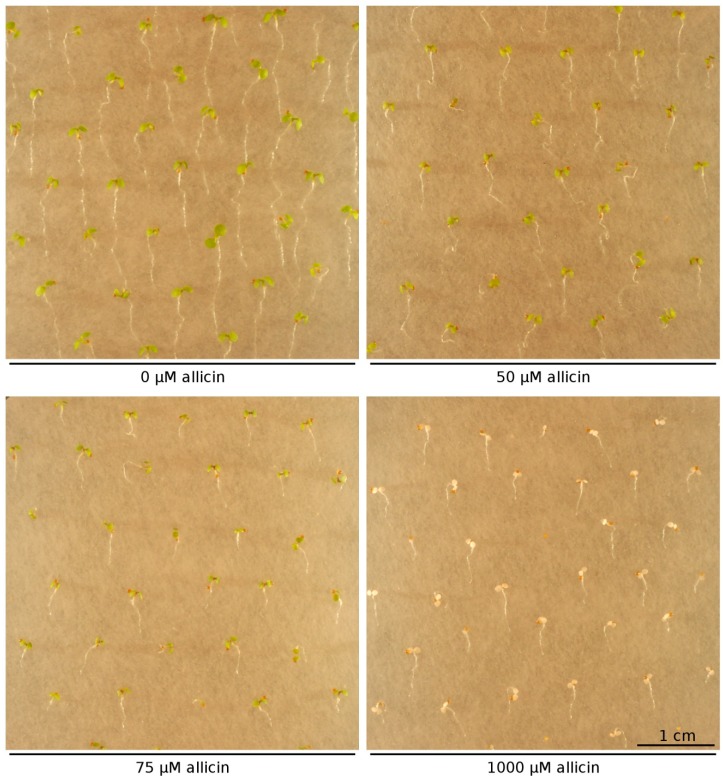
Effect of allicin on seedling development: *A. thaliana* (Col-0) wild type 48 h after allicin treatment. In this experiment, seeds were set on sterile filter papers which were placed on Murashige & Skoog (MS) medium. After stratification at 4 °C for 48 h, germination and growth took place with the Petri plates tilted to an angle of approx. 20° from vertical to ensure unidirectional root growth according to root gravitropism. After three days of cultivation, filter papers were transferred to MS media that contained different amounts of allicin. Pictures were taken 48 h after treatment. For the sake of clarity only a small representative fraction of seedlings is shown for relevant concentrations.

**Figure 4:**
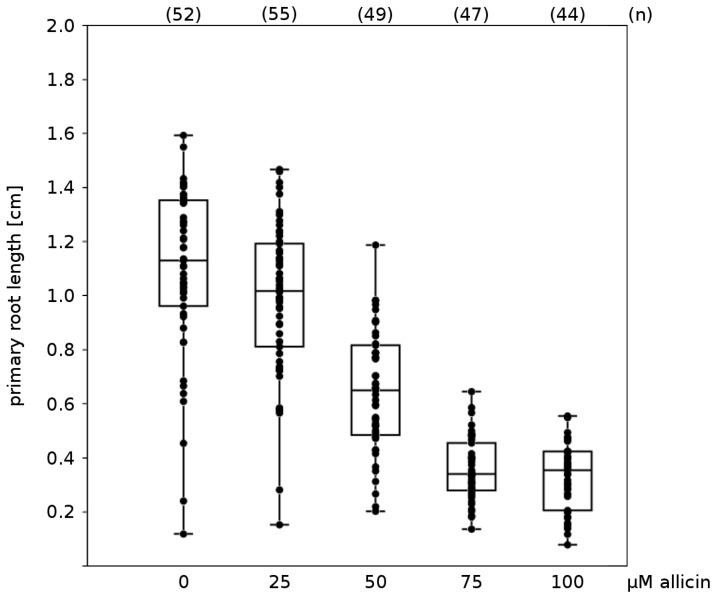
Effect of allicin on elongation of primary root growth: To investigate the effects of allicin on *A. thaliana* seedlings (accession Col-0), seeds were sown onto sterile filter paper which had been placed on Murashige & Skoog (MS) medium beforehand. After stratification (4 °C, 48 h), the Petri plates were cultivated at an angle approx. 20° from vertical to ensure unidirectional gravitropic root growth. For allicin exposure, the filter papers were transferred onto allicin-containing medium and 48 h after further cultivation, pictures were taken and the root lengths were measured using the software ImageJ [114]. The “boxplot” was prepared with the program PAST [115]; every dot represents the measurement of one root. The two halves of one box represent the 25 and 75 percent quartile, while the horizontal line in the middle shows the median. n = total number of measurements for the given treatment.

Two morphological effects could be seen by the naked eye (Figure 3). Firstly, exposure to allicin correlated with a decrease in primary root elongation in a concentration-dependent manner and the minimal allicin concentration needed to inhibit root elongation completely was determined to be 75 µM allicin (Figure 4). The fact that the root growth is inhibited by allicin without killing the whole plant indicates that root functions like the uptake of water and nutrients are presumably still operational.

**Figure 5:**
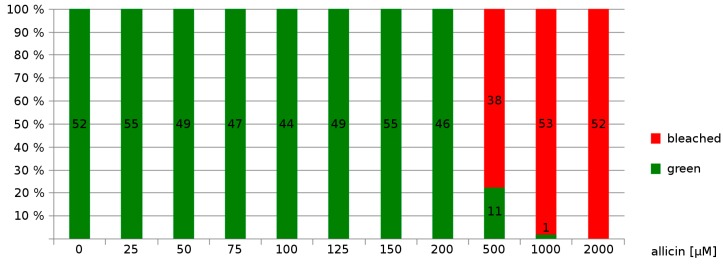
Allicin causes bleaching of seedlings: Sterile filter papers were placed on Murashige & Skoog (MS) medium and were sown with Col-0 *A. thaliana* wild type seeds. Following stratification (4 °C, 48 h), seeds were allowed to germinate and grow for three days. For allicin treatment, the filter papers were transferred onto MS medium containing different amounts of allicin. Effects on the seedlings were recorded 48 h after allicin treatment. Bleached seedlings showed either partial or complete bleaching, whereas green seedlings showed no signs of bleaching.

Secondly, exposure to a much higher concentration (500 µM allicin) led to bleaching of the seedlings (Figure 5).

**Figure 6:**
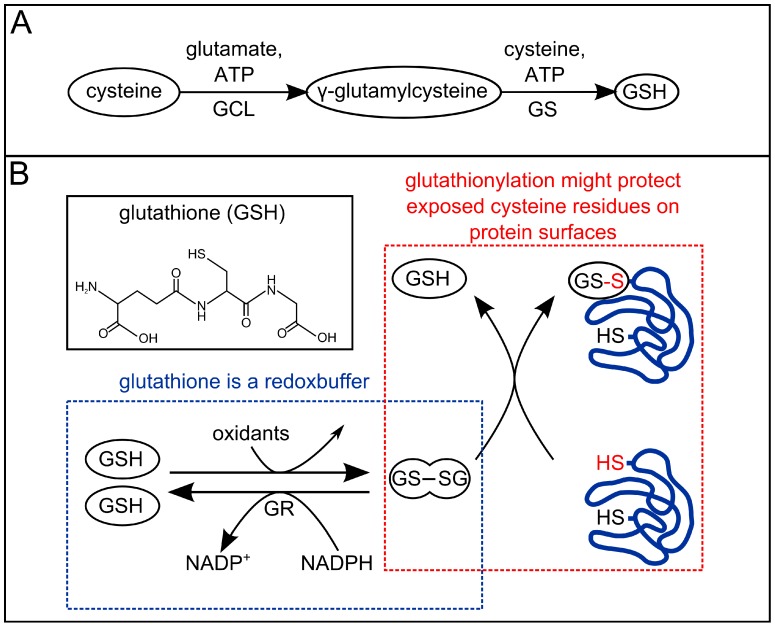
Glutathione biosynthesis and function: (**A**) Cysteine and glutamate are enzymatically linked to γ-glutamylcysteine via the action of glutamate cysteine ligase (GCL). The next step in synthesis is the formation of glutathione itself from cysteine and γ-glutamylcysteine via glutathione synthetase (GS). (**B**) Glutathione can be oxidized to glutathione dimers (GSSG) and thereby serves to buffer oxidants within the cell. A shift in the equilibrium from GSH towards a higher proportion of GSSG may promote the glutathiolation of free cysteines on protein surfaces, thus shielding these cysteine residues during oxidative stress conditions from over-oxidation. Illustration was redrawn from [116].

Because allicin is an efficient thiol reagent, we reasoned that a plant with reduced amounts of GSH should be more severely affected in the inhibition of seed germination and primary root growth than wild type (wt) plants. Therefore, we tested the *A. thaliana phytoalexin deficient 2-1* (*pad2-1*) mutant (Col-0 background), which only has approximately 20% of normal GSH levels due to a mutation in the gene coding for the enzyme glutamate cysteine ligase (GCL, Figure 6A) [112]. Seed-germination of the *pad2-1* mutant was found to be approximately 3× more sensitive to allicin than the wt and primary root growth was also inhibited at lower allicin concentrations than in the wt (data not shown). The greater sensitivity of the GSH-deficient *pad2-1* mutant is in accordance with earlier studies of *in vitro* enzyme inhibition by allicin [51,52,113]. Enzyme inhibition by allicin could be competed out by incorporation of cysteine or GSH into the reaction buffer and post-incubation with GSH led to a reversal of the inhibition for most (but not all) enzymes (*ibid.*). Although the enzyme inhibition studies were done *in vitro*, GSH is the most important and most abundant redox buffer *in vivo* in most eucaryotic and procaryotic cells [67], lending support to the idea that GSH is a protective agent against allicin *in vivo*.

Besides the fact that GSH can reduce disulfides back to thiols (Scheme 3), glutathiolation of accessible thiol groups can protect against their over-oxidation to sulfinic and sulfonic acids [23] (Figure 6B). As already discussed above, GSH is known to buffer the redox state of the cell [67]. Since it has been demonstrated that allicin also can shift the cell’s redox potential by oxidizing the glutathione pool, and push cells into apoptosis [68], the importance of GSH levels in cellular protection against allicin becomes doubly clear.

## 7. Conclusions

Allicin is a natural product which is consumed widely in most cultures that employ garlic in their diet. It is a physiologically active molecule with many potential health benefits. Furthermore, its potential for development in human and veterinary medicine is great and the concept of farmers being able to “grow their own” plant protection in an environmentally friendly manner is attractive, particularly in subsistence agriculture where the cost of commercial preparations may be prohibitive. This review, hopefully, will help to bring some of the scientific literature on the effects of allicin to the attention of interested scientists who may then further contribute to our understanding of this fascinating molecule.
